# Comparative Metatranscriptomics of Wheat Rhizosphere Microbiomes in Disease Suppressive and Non-suppressive Soils for *Rhizoctonia solani* AG8

**DOI:** 10.3389/fmicb.2018.00859

**Published:** 2018-05-04

**Authors:** Helen L. Hayden, Keith W. Savin, Jenny Wadeson, Vadakattu V. S. R. Gupta, Pauline M. Mele

**Affiliations:** ^1^Department of Economic Development, Jobs, Transport and Resources, Agriculture Victoria Research, AgriBio, Bundoora, VIC, Australia; ^2^CSIRO Agriculture and Food, Glen Osmond, SA, Australia; ^3^College of Medicine and Public Health, Flinders University, Bedford Park, SA, Australia; ^4^School of Applied Systems Biology, La Trobe University, Melbourne, VIC, Australia

**Keywords:** disease suppression, Rhizoctonia root rot, metatranscriptome assembly, differential gene expression, soilborne fungus, soil, rhizosphere, microbiome

## Abstract

The soilborne fungus *Rhizoctonia solani* anastomosis group (AG) 8 is a major pathogen of grain crops resulting in substantial production losses. In the absence of resistant cultivars of wheat or barley, a sustainable and enduring method for disease control may lie in the enhancement of biological disease suppression. Evidence of effective biological control of *R. solani* AG8 through disease suppression has been well documented at our study site in Avon, South Australia. A comparative metatranscriptomic approach was applied to assess the taxonomic and functional characteristics of the rhizosphere microbiome of wheat plants grown in adjacent fields which are suppressive and non-suppressive to the plant pathogen *R. solani* AG8. Analysis of 12 rhizosphere metatranscriptomes (six per field) was undertaken using two bioinformatic approaches involving unassembled and assembled reads. Differential expression analysis showed the dominant taxa in the rhizosphere based on mRNA annotation were *Arthrobacter* spp. and *Pseudomonas* spp. for non-suppressive samples and *Stenotrophomonas* spp. and *Buttiauxella* spp. for the suppressive samples. The assembled metatranscriptome analysis identified more differentially expressed genes than the unassembled analysis in the comparison of suppressive and non-suppressive samples. Suppressive samples showed greater expression of a polyketide cyclase, a terpenoid biosynthesis backbone gene (*dxs*) and many cold shock proteins (*csp*). Non-suppressive samples were characterised by greater expression of antibiotic genes such as non-heme chloroperoxidase (*cpo*) which is involved in pyrrolnitrin synthesis, and phenazine biosynthesis family protein F (*phzF)* and its transcriptional activator protein (*phzR*). A large number of genes involved in detoxifying reactive oxygen species (ROS) and superoxide radicals (*sod, cat, ahp, bcp, gpx1, trx*) were also expressed in the non-suppressive rhizosphere samples most likely in response to the infection of wheat roots by *R. solani* AG8. Together these results provide new insight into microbial gene expression in the rhizosphere of wheat in soils suppressive and non-suppressive to *R. solani* AG8. The approach taken and the genes involved in these functions provide direction for future studies to determine more precisely the molecular interplay of plant-microbe-pathogen interactions with the ultimate goal of the development of management options that promote beneficial rhizosphere microflora to reduce *R. solani* AG8 infection of crops.

## Introduction

Rhizoctonia root rot and bare patch disease, caused by the soilborne fungus *Rhizoctonia solani* anastomosis group (AG) 8, results in significant losses in cereal crops due to patches of stunted plants with reduced tillers and grain production (Hynes, 1933; Macnish, 1983; Rovira, 1986; Paulitz et al., 2010). In Australia yield losses are estimated to be $77 million per annum for wheat (*Triticum aestivum*) and barley (*Hordeum vulgare*) (Murray and Brennan, 2009, 2010). *Rhizoctonia solani* AG8 has a wide host range i.e., cereal crops, grasses, broadleaf crops, and weed species, but non-cereal crops in rotation have been shown to reduce pathogen inoculum levels (Gupta et al., 2012). However, in the absence of resistant cultivars of wheat or barley, current control measures are limited to management strategies such as cultivation to reduce *R. solani* AG8 hyphal networks, and herbicide application to remove inoculum carryover by weeds and volunteer plants (Roget, 1995; Roget et al., 1996). Fungicide seed dressings for pathogen control have previously proved ineffective or unreliable although a new product showing potential has been reported (Mckay et al., 2014; Almasudy et al., 2015). Therefore, a sustainable and enduring method for disease control is needed and may lie in the enhancement of biological disease suppression, where the resident microbial community counteracts the pathogen and/or restricts disease incidence (Cook et al., 1995; Wiseman et al., 1996; Roget et al., 1999).

In Australia and the Pacific Northwest of the USA, disease suppression has been demonstrated for *R. solani* AG8 and *Gaeumannomyces graminis* var. *tritici* (*Ggt*) (take-all disease) in cereal crops in farmer's fields and long-term trial sites, usually in association with continuous cereal cropping, stubble retention and no-till practices (Macnish, 1988; Roget, 1995; Pankhurst et al., 2002; Schillinger and Paulitz, 2006, 2014). Disease suppressive soils are defined as soils in which, because of their microbial composition and activity, a pathogen does not establish or persist, establishes but causes little or no disease, or establishes and causes disease for a while but thereafter the disease declines with successive crops of a susceptible host, even though the pathogen may persist in the soil (Cook and Baker, 1983; Schlatter et al., 2017). Soils with these characteristics have been identified globally for various soilborne plant pathogens including *R. solani* (Anees et al., 2010; Watanabe et al., 2011; Mavrodi et al., 2012), *Ggt* (Raaijmakers and Weller, 1998; McSpadden Gardener and Weller, 2001), *Plasmodiophora* spp. (Hjort et al., 2010), *Streptomyces* spp. (Sagova-Mareckova et al., 2015), *Thielaviopsis basicola* (Kyselková et al., 2009), *Pythium ultimum* (Löbmann et al., 2016), and *Fusarium oxysporum* (Alabouvette, 1986).

Various microbial mechanisms of disease suppression have been proposed. These include competition for sites and nutrients, particularly in the rhizosphere; antagonism via microbial production of volatiles, extracellular lytic enzymes and secondary metabolites such as iron-chelating siderophores and antibiotics; hyperparasitism; and elicitation of induced systemic resistance (ISR) by rhizobacteria (Weller et al., 2002; Raaijmakers et al., 2009; Pieterse et al., 2014; Schlatter et al., 2017). Antibiosis is the most studied mechanism of disease suppressive soils, where the growth and/or activity of one organism is inhibited by another organism via the production of specific or non-specific metabolites (Gómez Expósito et al., 2017). Antimicrobial producing bacteria, in particular *Pseudomonas* spp. capable of producing 2,4-diacetylphloroglucinol (DAPG), have received considerable attention because of their roles in suppression of black root rot of tobacco (Stutz et al., 1986; Ramette et al., 2006) and take-all of wheat (Raaijmakers and Weller, 1998; Cook, 2006). Latz et al. (2012) showed a correlation between disease suppression of *R. solani* (AG2–2IIIB) in sugarbeet seedlings and the abundance of two antibiotic genes *phlD* and *prnD* which produce the antifungal compounds DAPG and pyrrolnitrin respectively. Also being considered is the role of antimicrobial compounds at sub-inhibitory concentrations in modifying microbial community activity through intercellular signalling, motility, stress response, and biofilm formation (Romero et al., 2011; Philippot et al., 2013). Volatile compounds have been proposed to have a role in disease suppression as they may allow communication and competition between physically separated soil microorganisms, resulting in antagonistic interactions and changes in antibiotic and secondary metabolite production (Garbeva et al., 2014; Tyc et al., 2017). For most suppressive soils the mechanisms of suppression have not been fully defined (Schlatter et al., 2017). It has proven difficult to identify the mechanisms by which microorganisms suppress disease for many plant pathogens, possibly because of the multitude of interactive factors associated with crop type and cultivar, soil type, environment, and agronomic management practices, as well as the possibility of multiple organisms being involved. The microbial mechanism or mechanisms responsible for the suppression of *R. solani* AG8 are currently unknown.

The diversity of microorganisms involved in the suppression of some plant pathogens has been elucidated through the use of DNA-based community profiling techniques such as sequencing or microarrays (Kyselková et al., 2009, 2014; Sanguin et al., 2009; Mendes et al., 2011; Klein et al., 2013; Cha et al., 2015), including for *R. solani* AG8 suppressive soils (Yin et al., 2013; Donn et al., 2014; Penton et al., 2014). By contrast, the use of metagenomics to characterise the community genetic potential and metatranscriptomics to identify the expressed genes (mRNA) and active metabolic processes of microbial communities in suppressive soils remains novel and presents a significant opportunity to identify the complex microbial mechanisms. Two previous studies have examined disease suppression mechanisms using metagenomic or metatranscriptomic approaches. Hjort et al. (2010) focussed on the diversity of an antifungal chitinase gene in metagenomic DNA extracted from soil suppressive to club root disease of cabbage. In a metagenomic examination of the rhizosphere of sugar beet seedlings grown in pots, the introduction of *R. solani* AG2-2IIIB to a suppressive soil did not result in any change in bacterial composition, however metatranscriptomic analyses did reveal upregulation of stress related genes for oxidative stress response and guanosine-3,5-bispyrophosphate (ppGpp) metabolism in specific bacterial families (Chapelle et al., 2015). Based on these findings Chapelle et al. (2015) proposed a model for disease suppression whereby *R. solani* secretes oxalic and phenylacetic acid during colonization of the roots which exerts oxidative stress in the rhizobacterial community and the plant. This in turn may activate enhanced motility, biofilm formation and the production of yet unknown secondary metabolites by rhizosphere microbes to stop plant infection.

In this field-based experiment we examine the rhizosphere microbiome because it represents the frontline of defence for plant roots against attack by soilborne pathogens (Cook et al., 1995; Bowen and Rovira, 1999), and used a field site at Avon, South Australia that is historically well characterised for *R. solani* suppression (Roget, 1995; Roget et al., 1996; Barnett et al., 2006; Gupta et al., 2011; Donn et al., 2014; Penton et al., 2014). The objectives of this study were to use comparative metatranscriptomics (RNASeq) to characterise the functions and taxonomy of the rhizosphere microbiome in soils which are suppressive and non-suppressive for *R. solani* AG8, and identify differentially expressed microbial functional genes which may play a mechanistic role in disease suppression of *R. solani* AG8. Two different bioinformatic approaches were used to examine the rhizosphere samples based on unassembled and assembled analyses of the metatranscriptome libraries, and we report on differences in their annotation and differential expression analysis.

## Materials and methods

### Site details and management regimes

Two adjacent fields were studied on a farm in Avon, South Australia, in the southern wheat cropping region of Australia. The fields were suppressive (34°13′58.86″S, 138°18′35.16″E) and non-suppressive (34°14′23.70″S, 138°18′43.51″E) for the soilborne fungal pathogen *R. solani* AG8. The land at Avon has been utilised in field trials by the Commonwealth Scientific and Industrial Research Organisation (CSIRO) for over 25 years with the suppression of *R. solani* in cereal crops, resulting in reduced disease incidence, being well documented for more than 20 years (Roget, 1995; Roget et al., 1996; Barnett et al., 2006; Gupta et al., 2011; Donn et al., 2014; Penton et al., 2014).

The soil in both fields is classed as a Lithocalcic Calcarosol (Isbell, 2002). The climate is dominated by winter rainfall during the cereal cropping season and hot dry summers. Over the last 35 years the average total rainfall in the district was 338 mm per year, while the average growing season (April to October) rainfall was 253 mm (Bureau of Meteorology, Australia). For the 4 years prior to sampling, both fields were sown to cereals with wheat (*T. aestivum* cv. Gladius), barley (*H. vulgare* cv. Fleet), and oats (*Avena sativa* cv. Wallaroo) used in rotation. Both fields have been under continuous cereal cropping with stubble retention; though in the suppressive field this has occurred for more than 12 years compared to only 4 years in the non-suppressive field. Agronomic practices were similar for the two fields and comprised of direct drill sowing, fertiliser applications at sowing and 3 months after, and herbicide applications prior to sowing, during cropping and in the summer fallow period. In the year of sampling, both fields had been sown to the wheat variety Gladius on the same date (5 June 2012).

### Soil sampling

For each field, soil samples were collected 8 weeks post-sowing on 31 July 2012 when wheat was at tillering stage (GS20-29) and Rhizoctonia bare patches were visible in the non-suppressive field (Figure S1). There were four designated replicated 100 m^2^ plots in each field (GPS location described above) with two samples collected per plot, totalling eight soil samples per field. Each soil sample was made up of composited root+soil samples collected by digging up multiple wheat plants (~20) to a depth of 10 cm. All plants were collected randomly within the plot, hence samples included plants from both inside and outside of any disease patches. To collect soil samples, the root mass of each plant was gently shaken by hand. Soil that detached after gentle shaking was considered to be bulk soil, well homogenised for each sample, and eight samples were collected from each of the two fields for soil chemical and physical analyses (Table S1). Soil that was strongly adhered to the roots was considered as rhizosphere soil and detached by vigorous shaking of the root mass by hand. The rhizosphere soil was well homogenised for the samples and preserved in the field for RNA extractions. Eight rhizosphere samples each were collected from the disease suppressive and non-suppressive fields. Each rhizosphere sample for metatranscriptomics analysis was preserved for RNA extraction by adding 2 g of soil representing many different soil particles per sample to a tube containing 5 ml of Lifeguard (Mo-Bio, Carlsbad, CA, USA). The soil solution was shaken to make a suspension and then the tubes were kept at room temperature until our return to the laboratory where they were stored at −20°C. Bulk soil samples for chemical and physical analyses were stored at 4°C until they were shipped to a commercial laboratory (CSBP Lab, Bibra Lake, WA, Australia) for analyses according to National Association of Testing Authorities accredited standard procedures (http://www.nata.com.au).

Additional soil samples were also collected for the purpose of monitoring the abundance of *R. solani* AG8 inoculum in both fields. Samples were collected 1 week prior to sowing with a total of eight samples collected per field. Each sample was the product of 8 soil cores of 0–10 cm depth composited together, with two samples collected per replicate plot. Samples were also collected three times during the cropping season, using the rhizosphere sampling protocol described above, at 6 weeks post-sowing (plant disease incidence rating), 18 weeks post-sowing (physiological maturity), and 23 weeks post-sowing (harvest). The abundance of *R. solani* AG8 inoculum in both fields was determined by quantitative PCR (qPCR) as described in Penton et al. (2014). Briefly, DNA was extracted from each soil sample (~125 g) by the Root Disease Testing Service at SARDI (Adelaide, Australia) (Ophel-Keller et al., 2008). The qPCR assay was conducted using rDNA (TaqMan) probe sequences specific to *R. solani* AG8 (Ophel-Keller et al., 2008) and quantified against a standard curve of diluted *R. solani* AG8 genomic DNA (pg g^−1^ soil). Statistical significance of the difference in *R. solani* AG8 inoculum between suppressive and non-suppressive samples collected at the same time was tested using a paired *t*-test at *P* < 0.01.

### Root disease assessment

Plant samples were collected from both fields at 6 weeks post-sowing for Rhizoctonia root rot disease assessment. Twenty plants were collected for each of the 8 replicates per field, with two samples collected from each of the four designated replicated 100 m^2^ plots described above. Roots were carefully washed to remove adhered soil, scored for the incidence and severity of Rhizoctonia root rot disease using a 0–5 rating scale, and average percent disease index scores calculated (Mcdonald and Rovira, 1985). Statistical significance of the difference in the disease index (%) between suppressive and non-suppressive samples was tested using a paired *t*-test at *P* < 0.01.

### RNA extraction and cDNA sequencing

MoBio Lifeguard was removed from the thawed 2 g soil samples by centrifugation at 2,500 × g for 5 min. The solution was removed and the RNA extracted using an RNA PowerSoil Total RNA Isolation Kit (Mo-Bio). Extracted RNA was treated with DNase I (Turbo-DNA-free kit; Applied Biosystems, Foster City, CA, USA) to remove all traces of DNA. RNA quality and quantity was assessed using a NanoDrop 2000 spectrophometer (ThermoScientific) and gel electrophoresis.

Twelve samples (six suppressive and six non-suppressive) were used for mRNA enrichment using a Ribo-Zero Magnetic kit for Bacteria (specificity: 16S, 23S, and 5S rRNA from Gram-positive and Gram-negative bacterial total RNA) (Epicentre, Madison, WI, USA) according to manufacturer's instructions. A quantity of 1.65 μg of total RNA was used for each sample for mRNA enrichment. Ribosomal RNA-depleted RNA was cleaned by ethanol precipitation and the pellet resuspended in 18 μl of Elute, Prime, Fragment mix (TruSeq Stranded mRNA kit; Illumina, San Diego, CA, USA). Double-stranded cDNA was generated from the mRNA enriched RNA using Superscript II reverse transcriptase (Invitrogen, Carslbad, CA, USA) as per manufacturer's instructions. Library preparation using a TruSeq Stranded mRNA kit, processing and sequencing were performed in-house using the Illumina HiSeq 2000 with paired-end (PE) 100 bp sequencing of templates ~295 bp long. Metatranscriptomic libraries generated for all rhizosphere samples from the suppressive and non-suppressive fields (12 samples) are deposited in the NCBI Short Read Archive (study SRP126206; accession numbers SRR6349879–SRR6349890). The assembled metatranscriptome and Trinotate annotation file is available through Figshare (DOI:10.6084/m9.figshare.5657215).

### Bioinformatic analyses

**Figure 1:**
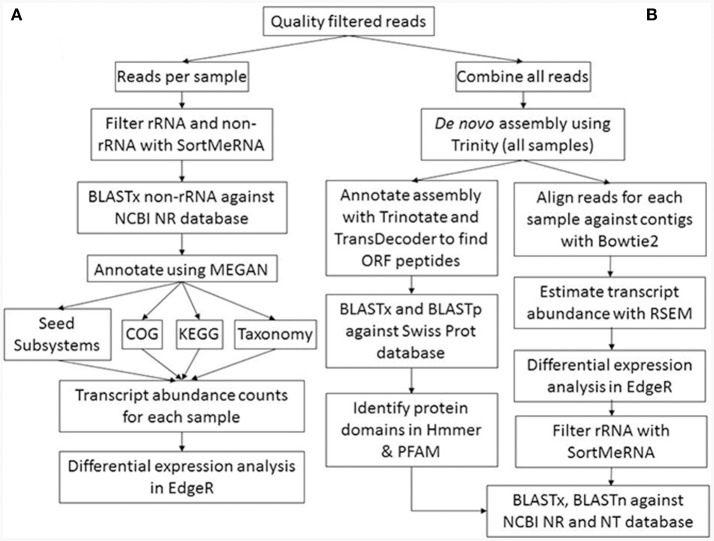
Metatranscriptome bioinformatic workflow. The two approaches used for metatranscriptomic analyses are shown: **(A)** direct annotation of short reads and differential expression analysis; **(B)** assembly of short reads into longer contigs, subsequent annotation, and differential expression analysis.

The raw Illumina paired-end sequence reads were quality filtered with the adaptors and low quality bases removed using Trimmomatic (Bolger et al., 2014). Default settings were used with the exception that minimum sequence length was set at 50 bp. Quality control of the filtered sequencing reads was performed by visual inspection using FastQC (http://www.bioinformatics.babraham.ac.uk/projects/fastqc). Two bioinformatic approaches were used to characterise the metatranscriptomes (see Figure 1 for the work flow). The first approach utilised unassembled transcripts to compare metatranscriptome libraries, while the second approach used was based on metatranscriptome assembly and undertaken to improve both annotation of transcripts and statistical analyses of differential expression of transcripts between suppressive and non-suppressive samples.

### Direct annotation of unassembled metatranscriptomic sequences

Despite mRNA enrichment, the remaining rRNA sequences were removed prior to BLASTX analyses of the filtered sequence reads. Ribosomal RNA removal was performed using the SortMeRNA software with the eight default rRNA databases included in the software package, covering the small (16S/18S), large (23S/28S), and 5/5.8S ribosomal subunit (Kopylova et al., 2012). The non-rRNA reads and rRNA reads were differentiated using the software with the non-rRNA reads considered to represent mRNA, and paired reads subjected to BLASTX searches (*E* ≤ 0.02) against the National Centre for Biotechnology Information (NCBI) non-redundant (nr) protein database to obtain the top five BLAST matches per sequence.

The BLAST results were parsed using the lowest common ancestor (LCA) algorithm in MEGAN (version 5.10.2) (Huson et al., 2007) with the default parameters. A comparison file was generated in MEGAN for all 12 samples using absolute read counts. The number of assigned reads per taxa was extracted for different NCBI-based taxonomy levels. For functional annotation, the protein coding sequences were classified using the Clusters of Orthologous Groups of proteins (COGs) database (Tatusov et al., 2000), Kyoto Encyclopaedia of Genes and Genomes (KEGG) database (Kanehisa et al., 2014), and SEED Subsystem database (Overbeek et al., 2014) with the number of assigned reads per functional group extracted for each sample.

### Metatranscriptome assembly and annotation

Trinity (version 2.2.0) (Haas et al., 2013) was used for *de novo* metatranscriptome assembly. The combined set of 348,722,194 quality filtered reads from all 12 rhizosphere libraries was combined into a single reference transcriptome assembly. Assembly and contig quality analysis was performed using both Trinity scripts and Transrate (Smith-Unna et al., 2016). The assembly was annotated using Trinotate (Bryant et al., 2017). Transcripts were subjected to a BLASTX search (*E* ≤ 1e^−5^) of the protein database Swiss-Prot (Boeckmann et al., 2005) downloaded from UniProt (http://www.uniprot.org/). The software Transdecoder (http://transdecoder.github.io) was used to predict likely coding regions within transcripts, and resulting protein products were subjected to a BLASTP search (*E* ≤ 1e^−5^) against the Swiss-Prot database. To identify conserved protein domains we used Hmmer software (http://hmmer.org/) and PFam (Finn et al., 2016). KEGG (Kanehisa et al., 2012), Gene Ontology (GO) (Ashburner et al., 2000), and Eggnog (Powell et al., 2012) annotations were retrieved from Swiss-Prot where transcripts could be assigned to these databases. All results for the reference assembly annotation were parsed by Trinotate, stored in a SQLite database and then reported as a tab-delimited summary file. The proportion of the assembly that represented rRNA reads was determined by aligning contigs to rRNA databases using Sort Me RNA as described above (Kopylova et al., 2012). Reads from each sample were individually mapped back onto the assembly using Bowtie (Langmead et al., 2009) and a count table of reads that align to each transcript produced using RSEM (RNA-Seq by Expectation-Maximization) (Li and Dewey, 2011) within the Trinity software package. Biological replicates were compared for the suppressive and non-suppressive metatranscriptome libraries using a Pearson correlation matrix computed in Trinity (Haas et al., 2013) based on transcript expression values.

### Differential expression analysis

For the unassembled annotation four count tables were generated in MEGAN for the assigned species, COG, KEGG, and SEED functions (Table S2). For the assembled metatranscriptome analysis one count table of the estimated transcript (isoform) abundance was generated using Trinity (Datasheet 1). All count tables were analysed in edgeR version 3.16.5 (Robinson et al., 2010) to identify significantly differentially expressed (DE) taxa, functional groups or transcripts using a *P*-value of < 0.05, a false discovery rate (FDR) cut-off of 0.05 and minimum fold change (FC) of 2 for the unassembled analyses and 4 for the assembled analyses. Data from the unassembled annotation in MEGAN were filtered at a count-per-million (CPM) of >100 because unassembled sequences could be aligned to multiple reference genes attributed to a taxon, COG, KEGG, or SEED group, and a taxon or functional group was only retained if it was expressed in at least three of the six replicate samples. For the assembled metatranscriptome, transcript abundance was filtered at a CPM of 0.5 as a “gene” grouping may contain a cluster of multiple transcripts (isoforms), though expression was required in five of the six replicate samples. Normalisation to allow comparison between samples was performed for each count table in edgeR using TMM (trimmed mean of *M*-values). EdgeR settings included using the generalised linear model (GLM) likelihood ratio test with the contrast option (suppressive minus non-suppressive) (McCarthy et al., 2012). Differentially expressed transcripts from the Trinity assembly were also subject to BLASTX and BLASTN searches of Genbank (*E* ≤ 1e^−5^) for comparison to MEGAN analyses.

## Results

### Rhizoctonia inoculum and root rot assessment

**Figure 2:**
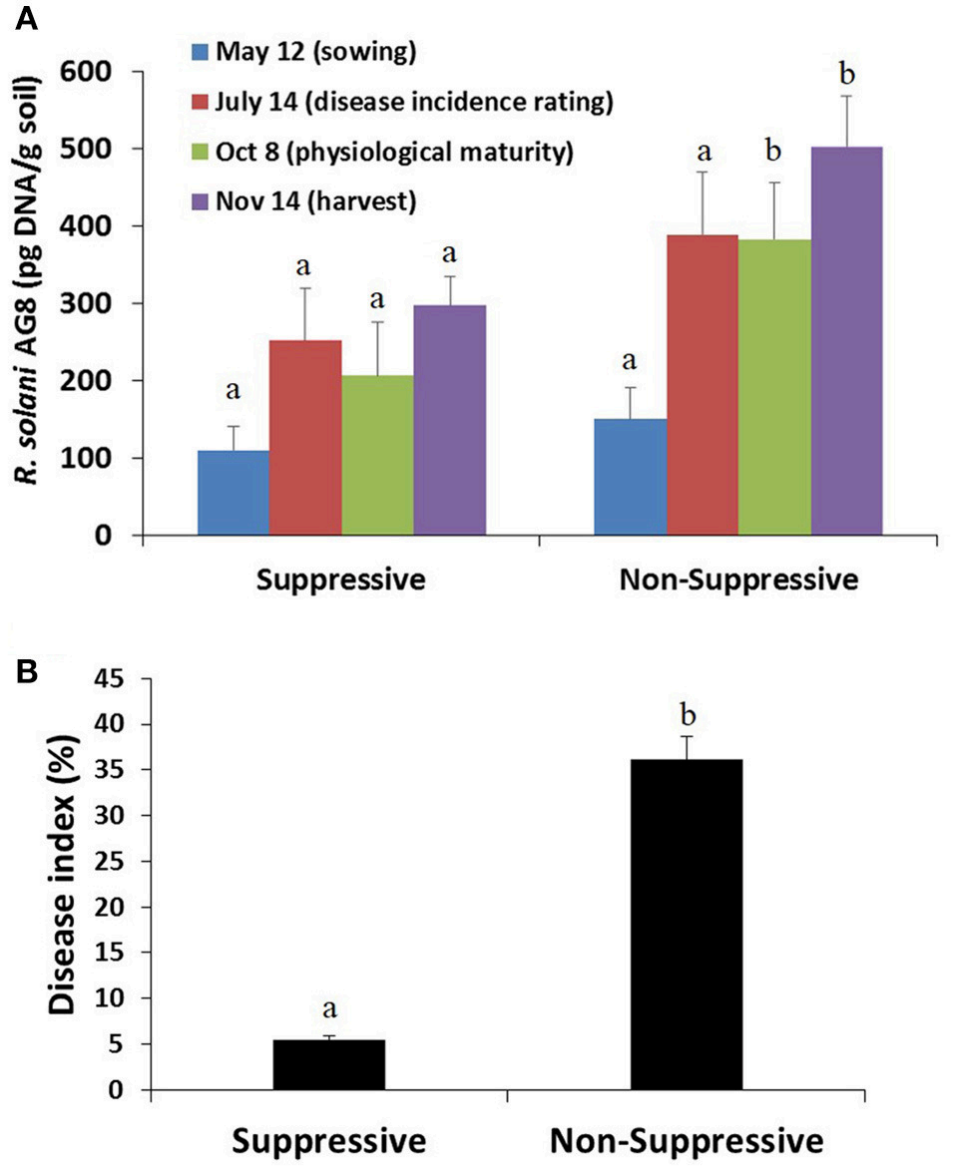
**(A)** Abundance of inoculum of the plant pathogen *R. solani* AG8 (pg DNA/g soil) as determined by quantitative PCR on soil samples collected prior to sowing and at different stages of the cropping season, and **(B)** root disease index (%) as assessed on plant roots sampled at 8 weeks post-sowing. Bars represent average values ± standard error. Values for suppressive and non-suppressive samples from the same sampling time that differed significantly (by paired *t-*test *P* < 0.01) are denoted with different letters.

Soils collected prior to sowing showed similar inoculum levels of the pathogen *R. solani* AG8 in both the suppressive and non-suppressive fields, as determined by quantitative PCR (pg DNA g^−1^ soil) (Figure 2A). These inoculum concentrations were considered a high disease risk condition. A build-up of inoculum was observed in both fields throughout the cropping season, in particular during the initial 8 weeks of crop growth. The build-up was greatest however, in the non-suppressive soils compared to that in the suppressive soils, resulting in significantly higher *R. solani* AG8 DNA in the non-suppressive soils (Figure 2A). These differences aligned with the data for the relative abundance of *R. solani* AG8 fungal transcripts expressed in the metatranscriptomic libraries from the rhizosphere samples collected at 8 weeks post-sowing (Figure S3).

An analysis of root samples from plants collected 8 weeks post-sowing showed very little Rhizoctonia disease incidence in the suppressive field (i.e., <5% infected roots with low level of infection), whereas the disease incidence and severity were significantly higher in plants from the non-suppressive field (i.e., >35% roots with higher level of infection) (Figure 2B). In the non-suppressive field disease incidence and infection of new roots, in particular crown roots, continued throughout the growing season until physiological maturity whereas there was no disease incidence on the crown roots of the crop in the suppressive field. The reduced vigour of Rhizoctonia infected plants, particularly in the non-suppressive field, would have contributed to the difference in grain yield between the two fields at harvest. The grain yield for the 2012 crop was 2.35 ± 0.08 t/ha for the suppressive field and 1.625 ± 0.04 t/ha for the non-suppressive field.

### Basic statistics of metatranscriptomics sequence data

Across the 12 metatranscriptome libraries sequenced, the overall quality score of the raw sequence reads was very high with the majority of the reads averaging Phred scores of Q ≥ 30 (>77% for all samples). The metatranscriptomes generated a total of 200,313,684 and 148,408,510 quality filtered reads for the suppressive and non-suppressive samples respectively (Table 1).

**Table 1:** Statistics of the unassembled metatranscriptome libraries.

**Soil**	**Sample**	**Raw reads**	**Quality filtered reads**	**rRNA reads**	**Non-rRNA reads**
Suppressive	AV145	38,898,414	24, 888, 912(63.98)	16, 126, 421(64.79)	8, 762, 491(35.21)
	AV148	31,581,940	23, 816, 024(75.41)	10, 858, 756(45.59)	12, 957, 268(54.41)
	AV149	44,143,322	31, 853, 580(72.16)	18, 030, 939(56.61)	13, 822, 641(43.39)
	AV150	54,343,882	37, 785, 266(69.53)	21, 279, 915(56.32)	16, 505, 351(43.68)
	AV151	54,156,402	38, 769, 816(71.59)	20, 908, 640(53.93)	17, 861, 176(46.07)
	AV152	59,201,762	43, 200, 086(72.97)	13, 107, 654(30.34)	30, 092, 432(69.66)
Non-suppressive	AV153	33,454,762	24, 555, 156(73.40)	5, 445, 349(22.18)	19, 109, 807(77.82)
	AV154	29,357,044	21, 604, 314(73.59)	7, 316, 666(33.87)	14, 287, 648(66.13)
	AV155	22,162,156	15, 962, 376(72.02)	4, 340, 483(27.19)	11, 621, 893(72.81)
	AV156	28,545 250	22, 576, 956(79.09)	7, 217, 907(31.97)	15, 359, 049(68.03)
	AV158	38,318,758	27, 607, 954(72.05)	9, 333, 510(33.80)	18, 274, 444(66.20)
	AV160	48,152,692	36, 101, 754(74.98)	9, 251, 760(25.63)	26, 849, 994(74.37)

*Total RNA was extracted from 12 soil samples collected from the rhizosphere of wheat in fields which were suppressive and non-suppressive for the soilborne disease Rhizoctonia solani AG8. The percentage proportion of quality filtered reads are given in parentheses. The values were calculated in relation to the number of raw reads per sample. The proportions of reads derived from rRNA and non-rRNA using SortMeRNA were calculated in relation to the total number of quality filtered reads per sample*.

### Characterisation of sequences in the unassembled metatranscriptomic libraries

Reads that aligned to rRNA databases were identified and excluded from further analyses of the unassembled metatranscriptome libraries. The proportion of rRNA reads remaining in the total RNA after rRNA subtraction was greater in the suppressive samples (30–65% of reads) than the non-suppressive samples (22–34% of reads). After filtering to remove rRNA reads, 67,551,136 and 73,540,104 reads for the suppressive and non-suppressive samples respectively were considered as possible protein-coding transcripts (mRNA) and used for BLAST analyses (Table 2). Of the BLASTX processed transcripts, 16,789,984 (25%) from the suppressive samples and 26,693,540 (36%) from the non-suppressive samples could be assigned in MEGAN to a taxonomic rank, and functionally to KEGG, COG, and SEED categories. Rarefaction curves for absolute sequence numbers at species level indicated near-coverage saturation in the 12 samples with no difference based on whether samples originated from suppressive or non-suppressive fields (Figure S2).

**Table 2:** Unassembled BLASTX processed non-rRNA reads for each of the 12 metatranscriptome libraries were assigned to taxonomic and functional databases using MEGAN.

**Soil**	**Sample**	**BLASTX processed non-rRNA reads**	**Reads with no hits**	**Unassigned reads**	**Taxonomy assigned reads**	**KEGG assigned reads**	**COG assigned reads**	**SEED assigned reads**
Suppressive	AV145	8,600,600	6,688,963	308,526	1,603,111	531, 384	126,596	117,696
	AV148	11,616,025	8,154,234	478,705	2,983,086	1,180,839	350,139	226,796
	AV149	12,403,113	8,270,323	539,077	3,593,713	1,416,226	407,949	286,704
	AV150	12,076,710	8,260,283	503,195	3,313,232	1,221,381	375,679	302,961
	AV151	10,554,942	7,214,807	453,908	2,886,227	1,124,750	361,126	249,757
	AV152	12,299,746	9,423,725	465,406	2,410,615	784,536	179,595	174,889
Non-suppressive	AV153	13,731,510	8,789,817	653,822	4,287,871	1,613,293	628,337	290,770
	AV154	11,725,325	7,610,545	529,943	3,584,837	1,334,134	448,388	236,235
	AV155	11,471,857	6,974,415	561,896	3,935,546	1,506,368	488,523	299,664
	AV156	12,466,243	5,779,271	657,011	6,029,961	2,125,805	593,659	583,782
	AV158	10,696,481	6,300,453	534,665	3,861,363	1,483,534	575,621	329,783
	AV160	13,448,688	7,755,148	699,578	4,993,962	2,031,003	733,993	367,619

Metatranscriptomic sequences were assigned to their closest taxonomic relative in the NCBI taxonomy based on the protein coding regions for bacteria, archaea, and eukaryotes (Table S2). Transcripts of *R. solani* AG8 were detected for each rhizosphere sample, confirming the presence and activity of the wheat pathogen in the root zone. The *R. solani* transcripts were most abundant in samples from the non-suppressive field and represented on average 6% of fungal sequences for the non-suppressive samples and 2% for the suppressive samples (Figure S3).

**Figure 3:**
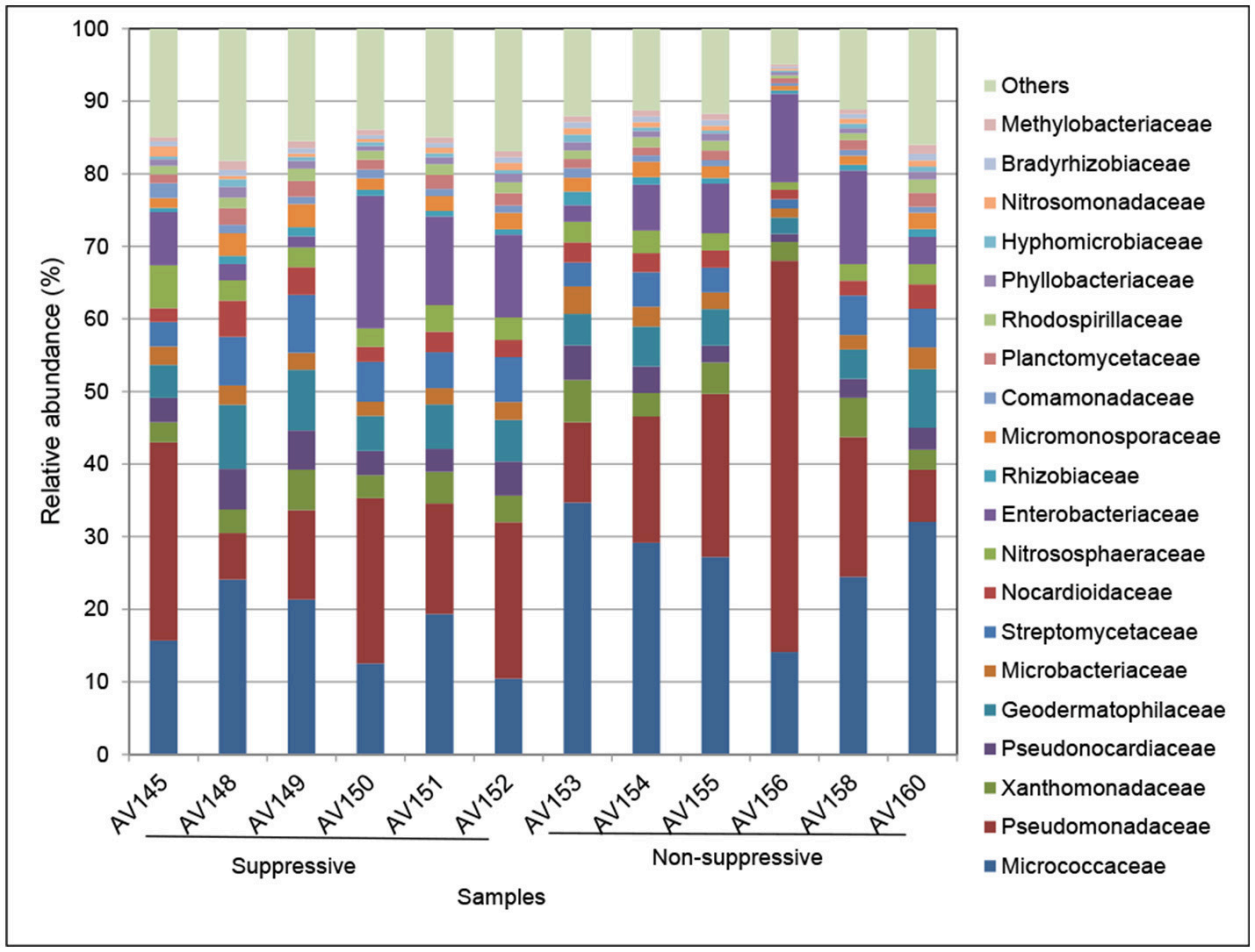
Relative abundance (%) of the major bacterial and archaeal families in metatranscriptomic libraries from rhizosphere samples collected from suppressive (AV145-AV152) and non-suppressive (AV153-AV160) soil. The taxonomic annotation is based on the Genbank non-redundant database and NCBI taxonomy. The category others represents families with a frequency of <1%, which included eukaryote transcripts.

The taxonomic composition of the active rhizosphere community based on mRNA sequences was very similar between the metatranscriptome libraries of the suppressive and non-suppressive samples. The rhizosphere community was primarily bacterial, dominated by the classes Actinobacteria, Alphaproteobacteria, and Gammaproteobacteria. The families Micrococcaceae (Actinobacteria), Pseudomonadaceae, and Enterobacteriaceae (Gammaproteobacteria) accounted for a large proportion of the rhizosphere microbiome and varied in abundance between individual samples (Figure 3). Archaea present in the rhizosphere were predominantly from the family Nitrososphaeraceae (phylum Thaumarchaeota). The dominant fungal phylum in the rhizosphere was Ascomycota, representing on average 72% of all fungal transcripts. Other active fungal phyla were Basidiomycota (10%), which includes the genus *Rhizoctonia*, and Glomeromycota (4%) which form arbuscular mycorrhizae. All other fungal transcripts belonged to five phyla present at <1% relative abundance or could not be identified below the rank of Kingdom (14%). Fungal families were a much smaller proportion of the total microbial transcripts in the rhizosphere though 164 fungal families were identified (data not shown).

**Figure 4:**
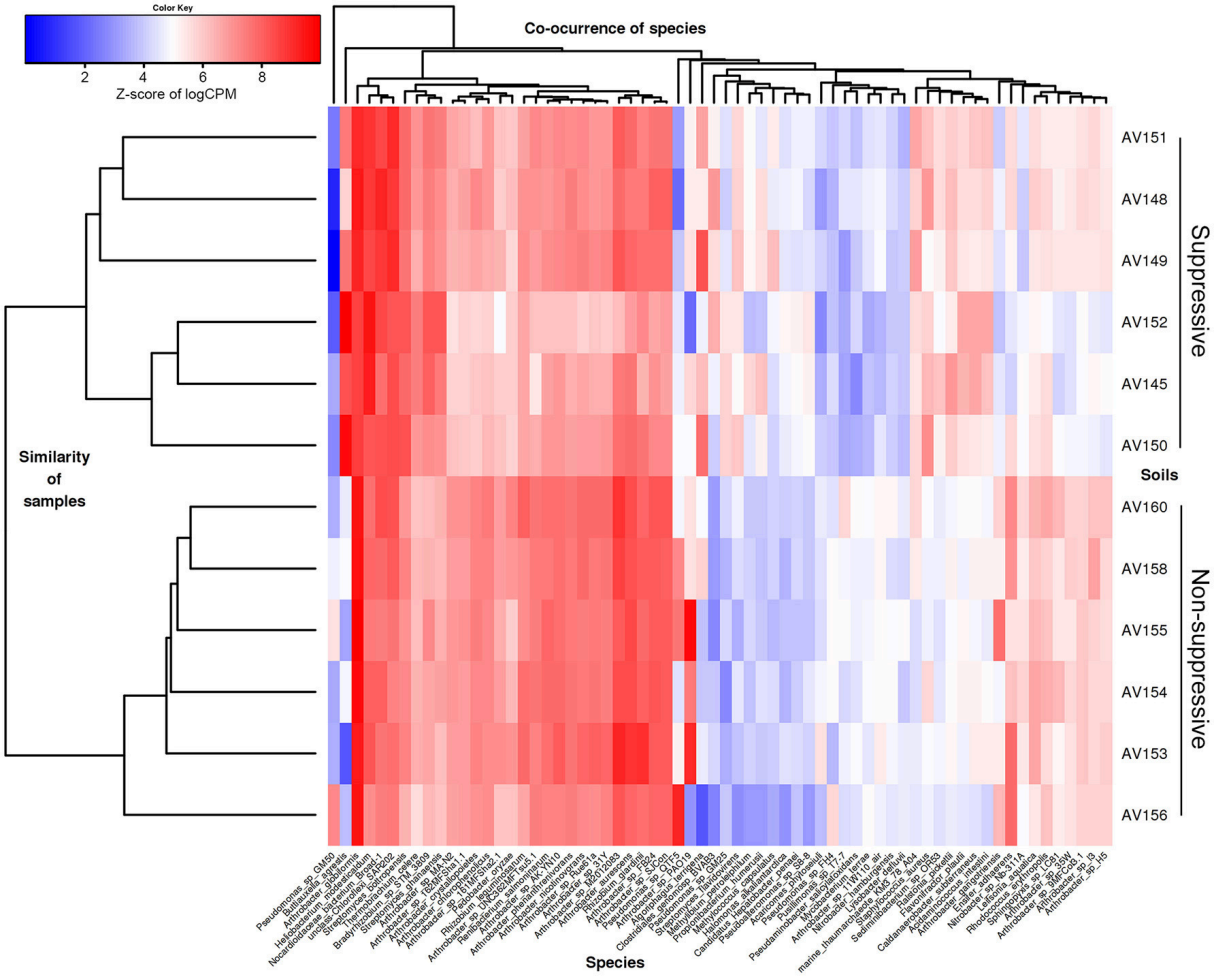
Heatmap showing microbial species with differential gene expression (FDR < 0.05, fold change > 2) for the unassembled metatranscriptomic libraries of suppressive (AV145-AV152) and non-suppressive (AV153-AV160) samples based on counts per million (CPM) sequence reads. Shown are species with the most extreme fold changes: logFC from −5.5 to −1 and 1 to 6.7.

Differential expression analysis at threshold of FC ≥ 2 showed that 65 bacterial species and one environmental archaeal strain differed significantly in their read counts (gene expression) between suppressive and non-suppressive fields (*P* < 0.05, FDR < 0.05; Figure 4, Table S3). Forty-two bacterial species from the Actinobacteria or Proteobacteria were more abundant (logFC = −1.044 to −5.491) in non-suppressive samples including 23 *Arthrobacter* species or strains (from NCBI taxonomy), three *Pseudomonas* spp., a *Rhizobium* spp., and a *Nitrobacter* spp. The 25 species more abundant (logFC = 1.050–6.669) in the suppressive samples were from a broad range of phyla including Actinobacteria, Proteobacteria, Firmicutes, Thaumarchaeota, Chloroflexi, and Bacteroidetes. The most abundant species in the suppressive samples based on fold change was *Buttiauxella* spp. (logFC = 6.669) while *Streptomyces* spp. were the most common with three species having significantly different expression (*P* < 0.05, FDR < 0.05, logFC = 1.195–2.249). Unlike the bacterial species, none of the fungal species detected in the metatranscriptome libraries showed significant differential expression between the suppressive and non-suppressive samples.

Functional profiling of the unassembled suppressive and non-suppressive libraries was undertaken by comparing the tentative functions assigned in the KEGG, COG, and SEED databases in MEGAN (Table S2). For KEGG analysis, transcripts for suppressive and non-suppressive samples were assigned on average to the dominant pathways of Metabolism (32%), Unclassified KEGGs (31%), Genetic Information (22%), Environmental information Processing (6%), or other categories (9%). The number of unique KEGG orthology (KO) identifiers found for all 12 samples was 10,669.

Differential expression analysis of KEGG functions revealed eight significant KEGG identifiers (*P*-value and FDR < 0.05, FC ≥ 2; Table 3). Four KO identifiers were more abundant in non-suppressive samples including two ABC transporter proteins (K02077, K02074), putrescine oxidase (K03343) which is an enzyme in the urea cycle and metabolism of amino groups, and glutaredoxin (K06191), an essential component of the glutathione system that reductively detoxify substances such as arsenic and peroxides and are important in the synthesis of DNA. The four KO identifiers more abundant in suppressive samples represented large and small subunit hydrogenases (K06281 and K06282 respectively), a Type VI secretion system secreted protein *Hcp* (K11903), and a surface adhesion protein (K12549).

**Table 3:** Identification of differentially expressed functional traits from the KEGG and COG databases with a fold change >1 in unassembled metatranscriptome libraries of suppressive samples compared to non-suppressive samples.

**KEGG (K) and COG/NOG^a^ identifiers**	**log_2_ FC^b^**	**log_2_ CPM^c^**	***P*-value**	**FDR^d^**	**Category 1**	**Category 2**	**Gene**	**Most common bacterial taxa**
NOG08625	−1.989	7.629	0.001	0.042	Poorly characterized	Function unknown	Hypothetical protein	Arthrobacter
COG1621	−1.584	8.880	0.002	0.049	Metabolism	Carbohydrate transport and metabolism	Glycosidase	Arthrobacter, Nocardiodes
NOG122331	−1.437	8.478	0.001	0.041	Poorly characterized	Function unknown	Hypothetical protein	Arthrobacter, Nocardiodes
K02077	−1.298	8.306	0.001	0.044	Structural complex/Transporters	Environmental information processing and ABC transporters, prokaryotic type	ABC transporter substrate-binding protein	Arthrobacter
K03343	−1.292	7.637	0.001	0.044	Metabolism	Amino acid metabolism	Putrescine oxidase	Arthrobacter
COG0340	−1.243	7.936	0.000	0.020	Metabolism	Coenzyme transport and metabolism	Biotin-(acetyl-CoA carboxylase) ligase	Arthrobacter
K06191	−1.162	7.482	0.000	0.022	Unclassified		Glutaredoxin	Arthrobacter
NOG06580	−1.007	7.782	0.001	0.042	Poorly characterized	Function unknown	Hypothetical protein	Arthrobacter, Nocardiodes
K02074	−1.004	7.481	0.000	0.042	Structural complex/Transporters	Environmental information processing and ABC transporters, prokaryotic type	ABC transporter ATP-binding protein	Arthrobacter
COG1319	1.004	7.444	0.001	0.048	Metabolism	Energy production and conversion	Molybdopterin dehydrogenase; carbon-monoxide dehydrogenase	Mix of species
COG2719	1.017	7.345	0.001	0.037	Poorly characterized	Function unknown	Stage V sporulation protein SpoVR	Mix of species
COG2718	1.044	7.241	0.000	0.034	Poorly characterized	Function unknown	Sporulation protein YhbH	Sporangium, Oscillochloris
COG3903	1.062	7.162	0.002	0.049	Poorly characterized	General function prediction only	XRE family transcriptional regulator	Mix of species
K06281	1.107	8.007	0.001	0.048	Metabolism	Xenobiotics biodegradation and metabolism	Hydrogenase, [Ni-Fe]-hydrogenase large subunit	Mix of species
COG3383	1.116	7.573	0.001	0.042	Poorly characterized	General function prediction only	Formate dehydrogenase subunit alpha	Mix of species
COG3696	1.208	8.884	0.000	0.007	Metabolism	Inorganic ion transport and metabolism	Acriflavin restistance protein	Mix of species
NOG14710	1.232	9.627	0.000	0.020	Poorly characterized	Function unknown	Zinc binding protein	Mix of species
COG1459	1.306	6.671	0.002	0.049	Cellular processes and signaling	Intracellular trafficking, secretion, and vesicular transport	Type II secretion, protein F	Mix of species
COG2805	1.414	7.388	0.001	0.042	Cellular processes and signaling	Intracellular trafficking, secretion, and vesicular transport	Twitching motility protein PilT	Mix of species
COG4796	1.519	6.585	0.002	0.049	Cellular processes and signaling	Intracellular trafficking, secretion, and vesicular transport	Type IV pilus secretin PilQ	Mix of species
K06282	1.581	8.756	0.000	0.015	Metabolism	Xenobiotics Biodegradation and Metabolism	Hydrogenase small subunit	Mix of species
COG3059	1.740	7.005	0.000	0.034	Poorly characterized	Function unknown	Inner membrane protein	Mix of species
K11903	1.788	9.943	0.001	0.044	Environmental Information Processing	Membrane Transport	Type VI secretion system secreted protein Hcp	Pseudomonas
K12549	2.050	6.628	0.000	0.026	Unclassified		Surface adhesion protein lapA	Pseudomonas
NOG243551	2.847	8.564	0.002	0.049	Poorly characterized	General function prediction only	General stress protein csbD	Flavobacterium

a *Non-supervised Orthologous Group (NOG)*.

b *Fold change. A negative FC means the functional trait is more abundant in the non-suppressive sample*.

c *Count per million*.

d *False discovery rate*.

For COG analysis, transcripts for suppressive and non-suppressive samples were assigned on average to the categories of Metabolism (35%), Information, Storage and Processing (28%), Cellular Processes (17%), and Poorly Categorised COGs (20%). The number of orthologous groups identified from the COG database for all 12 samples was 9,950. Seventeen orthologous groups showed differential expression (*P* < 0.05, FRD < 0.05, FC ≥ 2) (Table 3). The five COGs with greater transcript counts in the non-suppressive samples were glycosidase (COG1621) which is associated with carbohydrate metabolism, a biotin metabolism gene (COG0340) and three hypothetical proteins (NOG08625, NOG06580, NOG122331). The 12 COGs with greater transcripts counts in the suppressive samples had a variety of functions including sporulation protein *SpoVR* (COG2719), sporulation protein *YhbH* (COG2718), Type II secretion protein (COG1459), Type IV pilus secretin *PilQ* (COG4796), and twitching motility protein *PilT* (COG2805). Transcripts were assigned to 29 SEED pathways in MEGAN, though none of the SEED identified genes differed significantly in their expression between the suppressive and non-suppressive samples.

### Metatranscriptome assembly, annotation, and differential gene expression

We co-assembled libraries from 12 samples totalling 174,361,097 100 base paired-end reads. The assembly generated contained 2,092,555 transcript contigs clustered into 1,747,231 “gene” groupings totalling 620.7 Mb. The average transcript length was 296 bases, while 10% (N10) of contigs were 654 bases and 50% (N50) were 278 bases. The median contig length was 248 bases and the longest was 16,460 bases. The E90N50 contig length was 253 bases. Further assembly and contig quality statistics are provided in Table S4. The total number of original reads which mapped back to the assembly was 89% with almost all mapping as properly paired reads (87%). A comparison of biological replicates for the suppressive and non-suppressive libraries showed greater similarity between replicates from the same field than between fields (average Pearson correlation between replicates: 0.5 for suppressive and 0.43 for non-suppressive) and no outliers were identified in a Principal Component Analysis (PCA) (Figure S4). The number of contigs identified as rRNA subunits using Sort Me RNA was 106,746 (5.1%) while 1,985,809 contigs (94.89%) were identified as non-rRNA.

We applied Trinotate to the 2.1 million transcript contigs in our assembly, finding 886,783 transcripts matched 102,982 unique Swiss-Prot proteins, and 12,105 Trinity transcripts matched more than 80% of their length with the best matched protein sequence. The number of annotated transcripts was 960,807 (46%) when the additional databases of EGGNOG, KEGG, PFAM, and GO were considered as well as the Swiss-Prot BLAST annotations. Trinotate reported 9,055 unique KEGG identifiers, and 7,197 unique COG identifiers for the assembly. As with the unassembled MEGAN sequence analysis, transcripts of *Rhizoctonia* spp. (designated as *Thanatephorus* spp.*)* were detected in the metatranscriptome assembly, in this case as eight contigs representing the lignin-degrading enzymes laccase-1 and laccase-3.

For differential expression analysis, unassembled reads were aligned for each sample to the contigs to estimate the expression profiles of all contigs across the suppressive and non-suppressive samples (Data sheet 1). Analysis of the assembly revealed 7217 contigs with differential expression for suppressive and non-suppressive samples (*P* < 0.05, FRD < 0.05, FC ≥ 4). Of these differentially expressed contigs, 2,676 (37%) were rRNA subunits as they aligned with rRNA databases using SortMeRNA and BLASTN of NCBI nt (*E* ≤ 1e^−5^). Based on the BLASTN annotation, 63% of these rRNA contigs were bacterial, 34% were eukaryotic and <1% were archaeal. The majority of the rRNA contigs (2,360; 88%) were more abundant in the suppressive libraries while only 316 contigs (12%) were more abundant in non-suppressive libraries. After the removal of rRNA, 4541 differentially expressed contigs remained of which 2,652 were more abundant in non-suppressive libraries and 1,889 were more abundant in suppressive libraries.

A large number of the differentially expressed contigs could not be functionally annotated with the numerous functional databases used (BLASTX of protein databases NCBI nr and Swiss-Prot, BLASTP of Swiss-Prot, EggNog, KEGG, PFam, and GO) and were either hypothetical proteins or had no hits. Notably the number of these non-functionally annotated contigs was far greater for the suppressive (1,662; 88%) than non-suppressive samples (1,024; 39%). Of the DE contigs that could be functionally annotated 1,628 were more abundant in the non-suppressive libraries and 227 in the suppressive libraries (Table S5).

**Figure 5:**
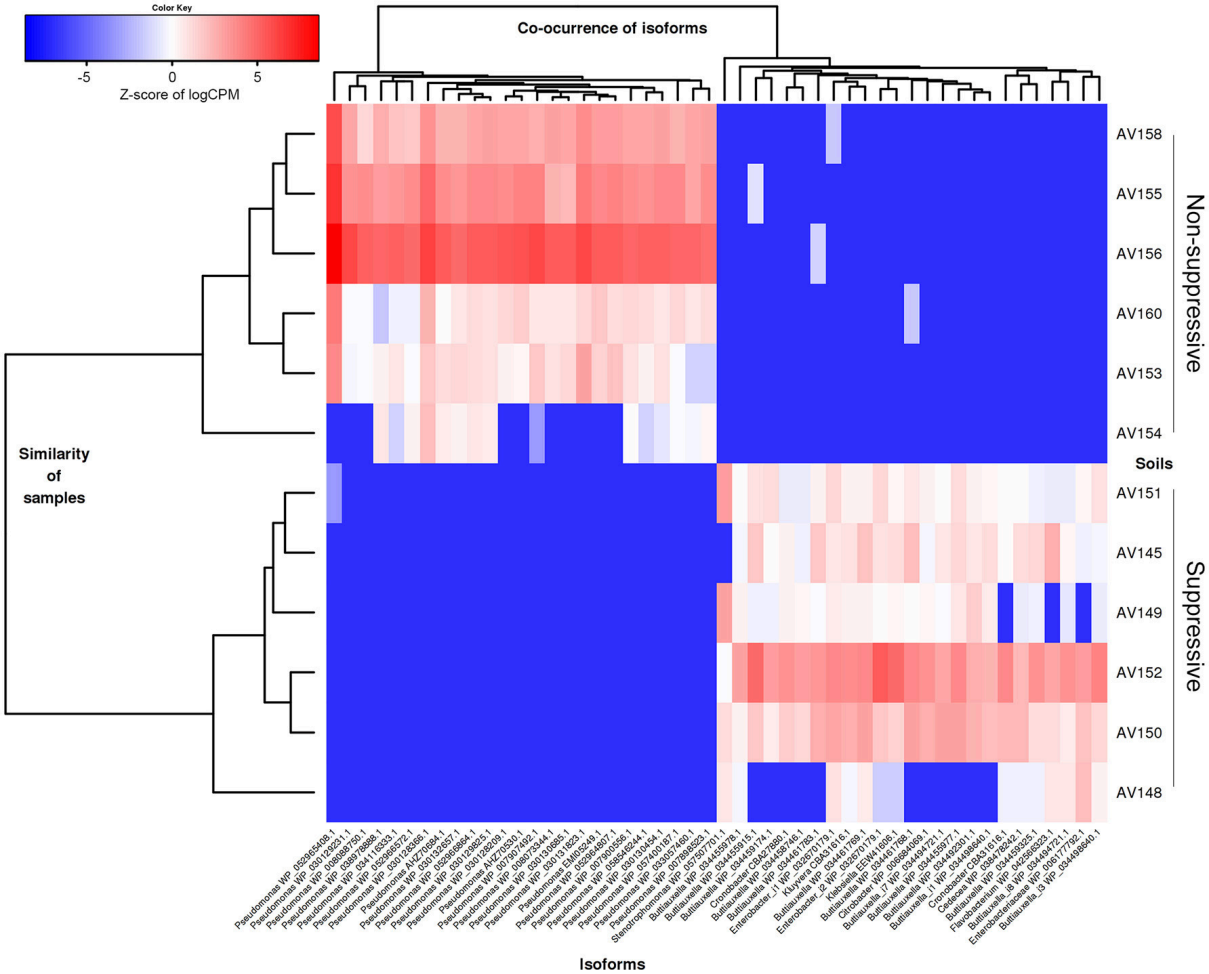
Heatmap showing the abundance of contigs (isoforms) based on their taxonomic annotation with differential gene expression (FDR < 0.05, fold change ≥ 4) for the metatranscriptomic libraries of suppressive (AV145-AV152) and non-suppressive (AV153-AV160) samples, based on counts per million (CPM) sequence reads. Contigs shown have the 25 highest and lowest fold changes (logFC from −15 to −11 and from +11 to +16 and FDR < 1e-08) and were able to be annotated at genus using the NCBI nr data with their Genbank identification number shown.

The taxonomic annotation of the DE contigs was examined using the BLASTX annotation of NCBI nr (as for the unassembled reads in MEGAN). The majority of contigs were bacterial (1499/1855 contigs; 81%) while archaea and eukaryotes represented <1% each (14 contigs) (Table S6). Contigs with greater expression in the non-suppressive libraries were from 76 genera with the most common species or strains being *Pseudomonas* spp. (593 contigs; 37%), *Arthrobacter* spp. (419 contigs; 26%), and *Stenotrophomonas* spp. (83 contigs; 5%) (Figure 5). Contigs with greater expression in the suppressive libraries represented only 30 genera though the frequency of the most common species or strains was much lower than in non-suppressive samples with *Stenotrophomonas* spp. the most common with 43 contigs (19%), followed by *Buttiauxella* spp. (33 contigs; 15%) and *Pseudomonas* spp. (10 contigs; 4%) (Figure 5). Based on BLASTP of Swiss-Prot 3% (62/1,855) of all annotated differentially expressed contigs were found to be viral (Table S5).

**Figure 6:**
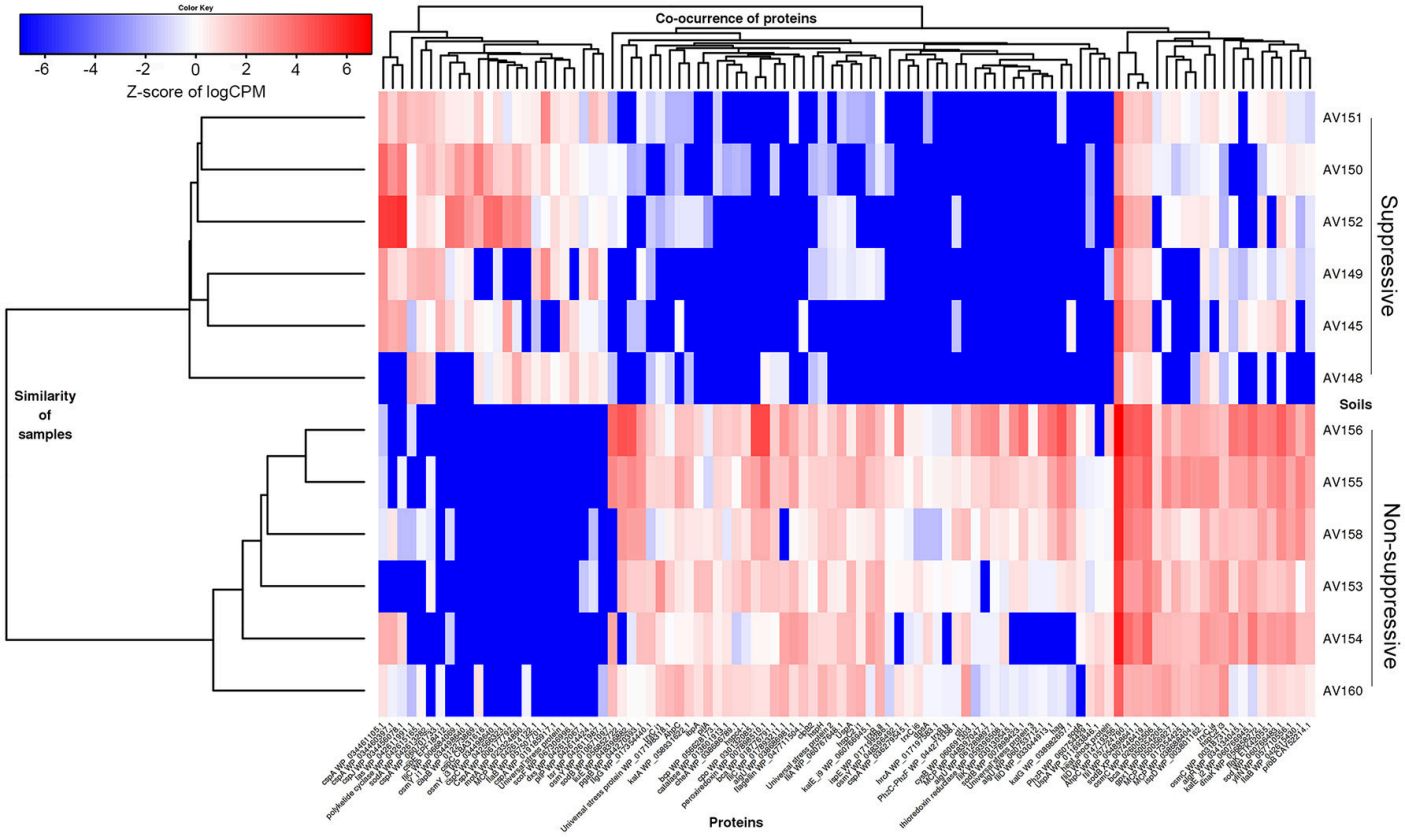
Heatmap showing differential gene expression (FDR < 0.05, fold change ≥ 4) for genes from the metatranscriptomic libraries of suppressive (AV145-AV152) and non-suppressive (AV153-AV160) samples, based on counts per million (CPM) sequence reads. A selection of contigs (isoforms) with rhizosphere related functions is shown annotated by gene name and the NCBI protein reference sequence number.

Functional characterisation of the DE contigs was considered using all database assignments. Of the 227 annotated contigs that were more abundant in the suppressive libraries (Table S5), a large number of contigs were involved in the functions of regulation of DNA transcription and ribosome translation. Genes outside of these functions that were expressed more in the suppressive samples included a polyketide cyclase, a terpenoid biosynthesis backbone gene 1-deoxy-D-xylulose-5-phosphate synthase (*dxs*), and many stress and adaptation genes with 11 contigs representing cold shock proteins (*csp, scoF, capB*) (Figure 6). Osmotic stress genes (*osmY*) were also expressed with two DE contigs being more abundant in suppressive samples and two in non-suppressive samples.

Functions of the 1,628 annotated DE contigs which were more abundant in the non-suppressive libraries (Table S5) included transcription, translation, carbohydrate metabolism, energy metabolism, lipid metabolism, and amino acid metabolism. Genes that were more abundant in the non-suppressive samples (Figure 6) included genes for the biosynthesis and regulation of antibiotics such as phenazine e.g., phenazine biosynthesis family protein (*phzf*) and its transcriptional activator protein (*phzR*), and pyrrolnitrin with non-heme chloroperoxidase (*cpo*). Also more abundant in the non-suppressive samples were five contigs for superoxide dismutase (*sod*) genes and 22 contigs representing genes for protection from reactive oxygen species (ROS) and superoxide radicals such as catalase (*kat, bca, osmC*), peroxiredoxins (*ahpC, bcp*), glutathione peroxidases (*gpx1, proB*), and thioredoxin system genes (*trx*). A large number of DE motility genes were more abundant in the non-suppressive samples than suppressive samples with 13 compared to three contigs for the genes flagellin (*fliC, fliA, fliK*), flagellar hook proteins (*flgK, fliD*), flagellar basal-body rod proteins and a fimbrial protein (*pilA*). Additionally, one contig for alginate biosynthesis genes (*alg*), which results in swarming motility, and two contigs for biofilm synthesis and regulation genes (*pgaB, bssS*) were expressed more in non-suppressive samples while the expression of chemotaxis genes (*mcp, motA, cheA, tas, tsr*) was similar for both suppressive and non-suppressive samples with four DE contigs each. While suppressive samples were characterised by greater abundance of contigs for cold-shock proteins, the non-suppressive samples displayed differential expression for stress response and adaptation with nine heat shock proteins (*ibpA, hsp*) and associated chaperones (*dnaK, clpB*), and a nutrient stress gene (*dspA*). Two contigs for genes in the metabolism of terpenoid backbone synthesis (*ispD, ispE*) were also expressed more in non-suppressive samples, forming a different part of the MEP/DOXP pathway to the gene expressed more in the suppressive sample (*dxs*). Interestingly, we also found a plant defence protein, chalcone synthase (*chs*), a type III polyketide synthase that was more abundant in the non-suppressive samples.

### Soil physico-chemical properties

Soil physico-chemical properties were measured for 16 bulk soil samples collected in the two fields (Table S1). The two fields were characterised by very similar soil textures with 7–11% clay, 8–12% silt, and 76–83% sand. Both soils were pH (water) 8.6. The suppressive soil had nearly twice the organic carbon (1.63%), total nitrogen (0.1%), and total P (0.04%) content of the non-suppressive field (0.94, 0.06, and 0.02% respectively).

## Discussion

Disease suppression of the cereal crop pathogen *R. solani* AG8 is regulated by resident soil microorganisms and may assist in the development of a complementary strategy for minimising damage to wheat crops. Central to this biocontrol strategy is the need to identify the mechanisms of suppression and microbes that mediate these functions. This study builds on the knowledge of disease suppression of *R. solani* AG8 at a long-term cereal cropping site at Avon, South Australia (Roget, 1995; Roget et al., 1996; Barnett et al., 2006; Gupta et al., 2011; Donn et al., 2014; Penton et al., 2014) using an approach that identifies the active functions of the suppressive and non-suppressive rhizosphere microbiome. The detection of DNA (by qPCR) and transcripts of *R. solani* in the rhizosphere of healthy wheat roots of the suppressive field at Avon supports the concept of disease suppression where the pathogen may be present but does not cause significant disease (Baker and Cook, 1974). Differential expression analyses revealed that suppressive rhizosphere soils contained more polyketide and cold-shock stress genes while non-suppressive rhizosphere soils expressed many different oxidative stress genes such as superoxide dismutase and peroxidases, as well as flagella and antibiotic biosynthesis genes for phenazine and pyrrolnitrin. Our study of a suppressive soil is the first to use the metatranscriptomic approach in a field crop.

Many of the active taxa in the metatranscriptomes were common to both the suppressive and non-suppressive rhizosphere soils (Figure 3). Of the taxa with differential expression, there were differences in the dominant genera and diversity of taxa between the two soils, based on the taxonomic affiliation with the NCBI nr database (Figures 4, 5). The active rhizosphere of non-suppressive soil was dominated by *Pseudomonas* spp. (Gamma-Proteobacteria) and *Arthrobacter* spp. (Actinobacteria). *Pseudomonas* spp. are highly prevalent rhizosphere bacteria (Doornbos et al., 2011) and are associated with plant growth promotion and disease suppression (Stutz et al., 1986; Weller et al., 2002, 2007; Raaijmakers et al., 2009). Our observation that *Pseudomonas* spp. transcripts are more abundant in the rhizosphere of non-suppressive soils agrees with reports of higher prevalence of *Pseudomonas* spp. in soils with greater take-all disease severity compared to suppressive soil (Sanguin et al., 2009), in soils within *R. solani* AG8 disease patches compared to outside patches (Donn et al., 2014), and on the surface of necrotic wheat roots infected with *Ggt* or *R. solani* compared to healthy roots (Chapon et al., 2002; De Souza et al., 2003). The increased the abundance of active *Pseudomonas* spp. observed in the non-suppressive field at Avon may be associated with the premature senescence of *R. solani* diseased roots in that field and the consequent nutrient release in the rhizosphere.

*Arthrobacter* spp. transcripts were also co-dominant in the rhizosphere of the non-suppressive soil compared to the suppressive soil. This genus is known for its plant growth promoting traits (Cacciari et al., 1971; Banerjee et al., 2010; Fernández-González et al., 2017), including antagonistic activity against plant pathogens such as *Fusarium* spp., *Pythium* spp., and *Verticillium dahliae* (Mohamed et al., 2017). Arthrobacter are a common soil bacterium with both oligotrophic and copiotrophic lifestyles (Fierer et al., 2007; Ho et al., 2017) and are known to occur in the rhizosphere of wheat (Landa et al., 2003; Lenc et al., 2015). The greater abundance of *Arthrobacter* spp. in the non-suppressive rhizosphere may reflect their ability to degrade plant cell wall components including cellulose, pectin and xylan (Fernández-González et al., 2017) as well as metabolising glucose and mannose (Fernández-González et al., 2017) which are common wheat root exudates (Baetz and Martinoia, 2014). The increased abundance of carbohydrate and amino acid metabolism genes in the non-suppressive samples supports the theory that *Arthrobacter* spp. may be utilising recalcitrant carbon substances from *R. solani* damaged roots, and along with *Pseudomonas* spp. be acting saprophytically to assimilate exudates from diseased roots.

To identify biological functional patterns that differed between the suppressive and non-suppressive soils and may be related to disease suppression we used differential gene expression analysis. Unique to this study were the large number of genes expressed in the non-suppressive samples in defence against oxidative stress (Figure 6). Expressed mostly by *Pseudomonas* spp. and *Arthrobacter* spp., were genes encoding superoxide dismutase which detoxifies superoxides, catalases and peroxidases which detoxify hydrogen peroxide (Levy et al., 1992) and thioredoxin system genes which donate electrons to peroxiredoxins to remove ROS and nitrogen species with a fast reaction rate (Lu and Holmgren, 2014; Figure 6). ROS have multiple roles in plant-pathogen interactions e.g., they may function in defence in a host plant through their direct toxicity to pathogens or the activation of various metabolic pathways, while necrotrophic pathogens such as *R. solani* can use oxidative processes during their attack and invasion of plant tissues (Waśkiewicz et al., 2014). The production of ROS in wheat roots infected by *R. solani* AG8 was reported by Foley et al. (2016), though they were unable to distinguish in the interaction whether the ROS originated from the pathogen or the host as both can produce and detoxify ROS. Interestingly, our study showed an overwhelming induction of ROS detoxifying genes (*sod, trx, kat, gpx, bca*, and *bcp)* occurred in the rhizosphere bacteria of the non-suppressive samples. Bacteria in nature are continuously challenged by toxic oxygen metabolites. *Pseudomonas* and *Arthrobacter* spp. may dominate in the non-suppressive rhizosphere under conditions of *R. solani* infection because they can survive and function by producing protective ROS detoxifying enzymes (Levy et al., 1992).

One of the most well characterised mechanisms of disease suppression is antagonism by the production of antibiotics (Raaijmakers and Weller, 1998; Weller et al., 2002; Ramette et al., 2006; Raaijmakers et al., 2009; Kinkel et al., 2012). Given this, we expected that any antibiotic genes detected in our metatranscriptome analyses would be associated with the suppressive samples. Instead we found three antibiotic genes from *Pseudomonas* spp. expressed more in the non-suppressive samples (Figure 6). These genes were for the production and regulation of phenazine (*phzf*, *phzR*) and pyrrolnitrin (*cpo*) and were 17–1,220 fold greater in the non-suppressive samples (Table S5). As we only sampled once in the cropping cycle for our metatranscriptome analysis we cannot exclude these antibiotics as having a role in *R. solani* suppression. The expression of phenazine and pyrrolnitrin genes by *Pseudomonas* spp. in the non-suppressive rhizosphere in this study could be part of a defence strategy to mediate competition between the highly abundant *Pseudomonas* and *Arthrobacter* species rather than as a defence against *R. solani* (Kinkel et al., 2012). The expression of antibiotic genes and oxidative stress may also be interlinked in *Pseudomonas* spp. as the mode of action of phenazine is the accumulation of hydrogen peroxide and superoxide (Mousa and Raizada, 2015), thus oxidative stress genes might be expressed endogenously.

Motility transcripts were another functional group with differential expression between the two rhizosphere soils, with 13 transcripts for flagella genes and an alginate biosynthesis gene expressed more in the non-suppressive samples compared to three flagella genes in the suppressive samples. The majority of the flagella genes were expressed by *Pseudomonas* spp. in the non-suppressive soil, and thus probably involved in colonisation of the rhizosphere. This process requires flagella-dependent motility for migration into the root zone, followed by bacterial biofilm formation (Mauchline et al., 2015).

In the suppressive soil the dominant differentially expressed taxa were *Stenotrophomonas* spp. (for assembled analyses only) and *Buttiauxella* spp, both Gamma-Proteobacteria, as well as diverse taxa including *Pseudomonas* and *Streptomyces* spp. (Actinobacteria) (Figures 4, 5). *Stenotrophomonas* spp. transcripts were the most abundant representing 19 and 5% of the contigs with greater differential expression in the suppressive and non-suppressive samples respectively. *Stenotrophomonas* spp. are of great interest because of their plant growth promotion traits and use as biocontrol agents (Berg et al., 2010; Schmidt et al., 2012). *Stenotrophomonas maltophilia* is commonly found in the rhizosphere of many plants (including wheat) or as endophytes (Berg et al., 1999) and has been shown *in vitro* to produce antifungal xanthobaccins and the macrocyclic lactam antibiotic maltophilin (Berg et al., 1996; Ryan et al., 2009). Furthermore *S. maltophilia* has also been shown to inhibit *P. ultimum*, both *in vitro* and in soil microcosms, by producing chitinase and protease (Dunne et al., 1997). Both *S. maltophilia* and *Stenotrophomonas rhizophila* can also produce volatile organic compounds (VOCs) which inhibit mycelial growth of *R. solani* on cabbage (Kai et al., 2007). *S. maltophilia* was found to be more abundant in the rhizosphere of soil suppressive of *R. solani* on sugar beet (Mendes et al., 2013), which aligns with our finding of greater expression of *Stenotrophomonas* spp. in soils suppressive of *R. solani* AG8. The *Stenotrophomonas* spp. contigs that were significantly more abundant in suppressive rhizosphere soil were identified as genes for chemotaxis, flagellin, fimbrial protein, polyketide cyclase, superoxide dismutase, and outer membrane proteins.

*Buttiauxella* was another dominant genus with greater differential expression in the suppressive rhizosphere (4 and 15% of suppressive soil DE genes for unassembled and assembled analyses respectively). Expressed genes of this genus were characterised by fold changes of up to 100 and 650 in the unassembled and assembled analyses respectively. *Buttiauxella* spp. have been isolated from soil and fresh water (Kämpfer, 2015) though little is known of their role in soil. Antimicrobial activity has been demonstrated for a glycolipid extracted and identified from a soil *Buttiauxella* sp, however the biosynthetic pathway of the glycolipid is currently unknown (Marzban et al., 2016). The dominance of *Buttiauxella* spp. in the suppressive rhizosphere may also be related to carbon substrate preferences. Goldfarb et al. (2011) showed that *Buttiauxella warmboldiae* str. DSM 9404 preferentially used the amino acid glycine as a labile carbon source for growth in soil microcosms and glycine is exuded by healthy wheat roots (Warren et al., 2016). The substantial difference in expression of genes of *Buttiauxella* spp between suppressive and non-suppressive rhizosphere soil requires further investigation.

*Streptomyces* spp. comprised a small proportion of the transcripts with greater abundance in suppressive samples (12% for unassembled analyses). This genus has been well documented as agents of disease suppression, biocontrol, plant growth promotion, and broad-spectrum antibiotic production (Liu et al., 1996; Weller et al., 2002; Bakker et al., 2010; Kinkel et al., 2012; Govindasamy et al., 2013; Schlatter et al., 2017). A small number of transcripts from *Pseudomonas* spp. were also differentially expressed more in the suppressive samples (4% for assembled and unassembled analyses), though these represented contigs from different gene families to those expressed in the non-suppressive samples.

To date unassembled read-based analyses are more frequently used for soil or rhizosphere functional metatranscriptome studies (Stewart et al., 2011; Damon et al., 2012; de Menezes et al., 2012; Nacke et al., 2014; Tveit et al., 2014; Yergeau et al., 2014; Chapelle et al., 2015; Hultman et al., 2015; Kim and Liesack, 2015; Malik et al., 2016; Newman et al., 2016; Masuda et al., 2017) than metatranscriptome assembly (Holmes et al., 2017), possibly because they are simpler to use. Our study identified that for the metatranscriptomes of suppressive samples the interpretation of differentially expressed genes was very different depending on whether the analyses were undertaken on unassembled or assembled reads. For the unassembled analyses, genes that were more abundant in the suppressive samples compared to the non-suppressive samples included the surface adhesion protein *lapA*, twitching motility protein *pilT*, and type II, IV and VI secretion proteins (secretion protein F, *pilQ, Hcp*, respectively) (Table 3). Type II secretion systems are involved in surface adhesion, colonisation, biofilm formation, genetic material uptake and virulence in mammalian and bacterial plant pathogens such as *Ralstonia solanacearum, Xylella fastidiosa*, and *Xanthomonas* spp. (Burdman et al., 2011) while Type VI secretion proteins have been shown to contribute to pathogenicity in many bacteria (Zhou et al., 2012). A potential association between secretion systems and disease suppression has been demonstrated for *Pseudomonas fluorescens* Pf29Arp and take-all disease (Marchi et al., 2013). The bacterium reduced the severity of take-all disease, though genome analysis revealed it lacked antibiotic synthesis gene clusters and expressed different patterns for type III and type VI secretion systems on healthy and *Ggt* diseased wheat roots (Marchi et al., 2013).

In the assembled analyses, differentially expressed genes that were more abundant in suppressive samples included a polyketide cyclase gene, a terpenoid biosynthesis gene (*dxs*), as well as cold shock (*csp, scoF, capB*), universal (*Usp*), and osmotic (*osmY*) stress proteins (Figure 6), however only 12% of these DE genes could be annotated, indicating there is still much we don't know about the active functions of the suppressive soil microbiome. Suppressive samples expressed more genes involved in the regulation of DNA transcription and ribosome translation than non-suppressive samples, as well as cold shock genes which have been implicated as transcription and translation factors (Pearson et al., 2015). Terpenoid transcripts were differentially expressed in the two rhizosphere soils though they represented different parts of the KEGG terpenoid backbone biosynthetic pathway (data not shown). Polyketides and terpenoids are both antimicrobial secondary metabolites (Mousa and Raizada, 2015), with the antibiotic polyketides DAPG and pyoluteorin known to be associated with disease suppression (Mousa and Raizada, 2015). Given the ability of *Stenotrophomonas* spp. to produce antibiotics and secondary metabolites such as VOC, both the genus and the polyketide cyclase gene expressed in our study provide good targets for further investigation of *R. solani* AG8 suppression mechanisms.

Garoutte et al. (2016) described an in-depth comparison of bioinformatic analysis approaches for a soil metatranscriptome using assembled and unassembled protocols though they primarily focussed on annotation and did not have replicates to test how annotation methods affect differential gene expression. An evaluation of the two bioinformatic approaches used in this study to characterise and compare the rhizosphere metatranscriptomes of suppressive and non-suppressive soils revealed that each approach had different advantages or disadvantages regarding annotation and differential expression analysis. The first approach of using unassembled reads in MEGAN was most beneficial in providing a visual and numerical comparison of the metatranscriptome annotations of individual samples, though functional annotations were limited to the built-in databases in the software. A disadvantage of the unassembled analysis was the computationally intense and time consuming BLAST processing required for sample annotation, though this issue has been addressed by the development of the software DIAMOND (Buchfink et al., 2015). Gene annotation for the unassembled analysis was possible for only 25 and 36% of transcripts for the suppressive and non-suppressive samples respectively. In comparison, our second approach of co-assembling the 12 metatranscriptomes into a single assembly allowed more databases to be used in annotation and resulted in 46% of contigs being annotated.

The assembly approach proved to be most advantageous when it came to discriminating differentially expressed genes in the suppressive and non-suppressive samples. The assembly-based analysis is contig-centric and resulted in 7217 DE contigs (FC ≥ 4), while the unassembled read-based approach is gene-centric and resulted in only 25 DE KEGG and COG identifiers at lower stringency (FC ≥ 2). Two explanations exist for this difference. Firstly, in the assembled analysis the statistical tests are done independently of the annotation while in the unassembled analysis only counts of annotated genes and taxa are used. Secondly, gene counts per sample are generally higher when obtained through MEGAN compared to mapping samples back to contigs. This is because a gene binned by MEGAN as an individual KEGG or COG identifier may represent multiple Genbank reference genes with different alignments along that gene. On the basis of our findings, where differential gene expression analysis of soil or rhizosphere metatranscriptomes is to be performed we recommend using a *de novo* assembly approach so that DE analysis is independent of the annotation, and the use of six or more replicate samples to improve the similarity of biological replicate metatranscriptomes for statistical analyses.

In a recent review of the soil microbiome of disease suppressive soils, Schlatter et al. (2017) suggested that for *Rhizoctonia* suppression, a unified theory for the mechanism responsible remains elusive. In a study of the rhizosphere metatranscriptome of sugar beet seedlings in a *R. solani* AG2-2IIIB suppressive soil inoculated with *R. solani*, Chapelle et al. (2015) found increased expression of oxidative stress genes and ppGpp metabolism by rhizosphere bacteria. Our field-based experimental design differed from this pot trial (Chapelle et al., 2015) and instead found evidence of multiple oxidative stress genes for the detoxification of ROS and superoxide radicals expressed in the rhizosphere of plants collected from a non-suppressive field, where symptoms of *Rhizoctonia* bare patch disease were present (Figure S1). In view of the complexity of gene functions uncovered in metatranscriptomic analyses and that Rhizoctonia infection of cereal crops occurs over long periods, i.e., seedling to physiological maturity (Gupta et al., 2012), we highlight the need for future targeted and temporally-based approaches to further investigate the role of specific differentially expressed microbial taxa and genes e.g., polyketide cyclases, that may be potential key mechanisms for the suppression of Rhizoctonia root rot disease. In summary, this field-based metatranscriptomic study has characterised the active functions and taxonomy of the rhizosphere microbiome in soils which are suppressive and non-suppressive for *R. solani* AG8.

## Author contributions

HH: Obtained funding for the study, conceived and designed the experiments, conducted field sampling, carried out the bioinformatic analyses of the metatranscriptomes and wrote the paper; JW: Carried out the RNA extractions and metatranscriptome library preparations; KS: Conducted the differential expression analyses in edgeR, NCBI BLAST analyses and edited the paper; VG: Contributed to the design of the experiments, conducted field sampling, analysis of the qPCR and disease assessment data, and contributed to the manuscript preparation; PM: Contributed to manuscript writing and editing.

### Conflict of interest statement

The authors declare that the research was conducted in the absence of any commercial or financial relationships that could be construed as a potential conflict of interest.
