# Supplementary material for: Comparative Metatranscriptomics of Wheat Rhizosphere Microbiomes in Disease Suppressive and Non-suppressive Soils for Rhizoctonia solani AG8
Source: Front Microbiol. 2018 May 4;9:859. doi: 10.3389/fmicb.2018.00859 (PMC5945926; doi:10.3389/fmicb.2018.00859)
Supplement: Supplementary file 8 [file Image_1.pdf]

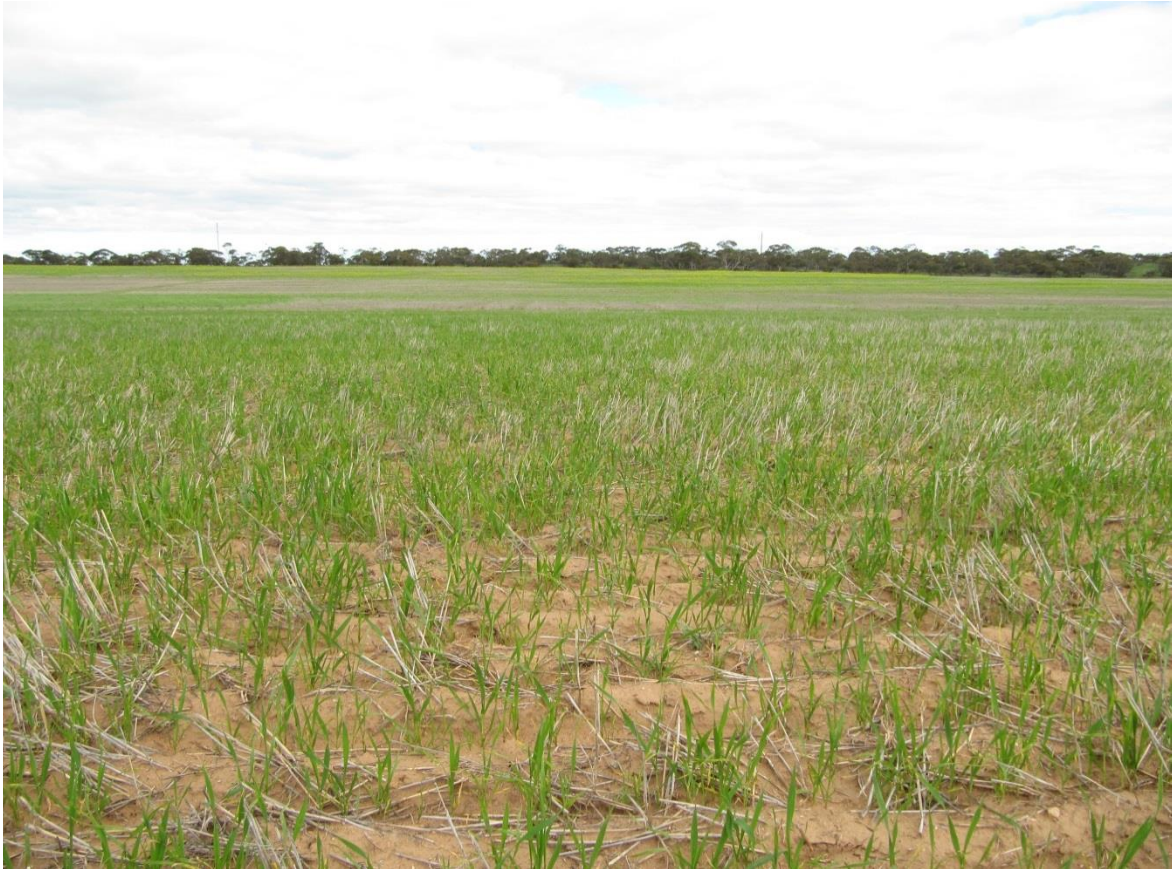

FIGURE S1. Rhizoctonia bare patch symptoms in the non-suppressive field at eight weeks post-sowing when rhizosphere samples were collected from both suppressive and non-suppressive fields. The barepatch in the foreground is a large open space in the crop canopy and plants show reduced vigour and tiller numbers. No patches were visible in the suppressive field which has a long history of *Rhizoctonia solani* AG8 suppression.
