# Supplementary material for: Comparative Metatranscriptomics of Wheat Rhizosphere Microbiomes in Disease Suppressive and Non-suppressive Soils for Rhizoctonia solani AG8
Source: Front Microbiol. 2018 May 4;9:859. doi: 10.3389/fmicb.2018.00859 (PMC5945926; doi:10.3389/fmicb.2018.00859)
Supplement: Supplementary file 9 [file Image_2.pdf]

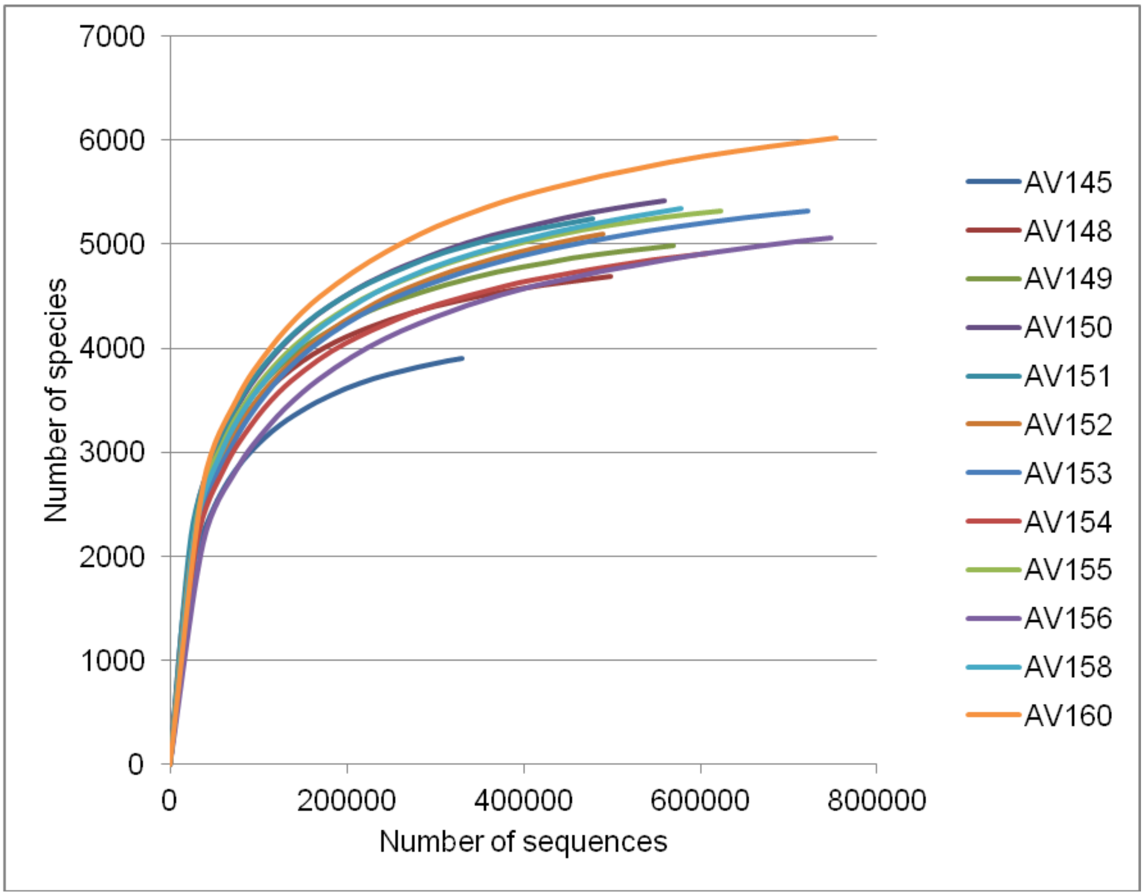

FIGURE S2. Rarefaction curves for mRNA sequences from suppressive (AV145-AV152) and non-suppressive (AV153-AV160) samples assigned at species level on the basis of NCBI taxonomy and lowest common ancestor hierarchy in MEGAN.
