# Supplementary material for: Comparative Metatranscriptomics of Wheat Rhizosphere Microbiomes in Disease Suppressive and Non-suppressive Soils for Rhizoctonia solani AG8
Source: Front Microbiol. 2018 May 4;9:859. doi: 10.3389/fmicb.2018.00859 (PMC5945926; doi:10.3389/fmicb.2018.00859)
Supplement: Supplementary file 10 [file Image_3.pdf]

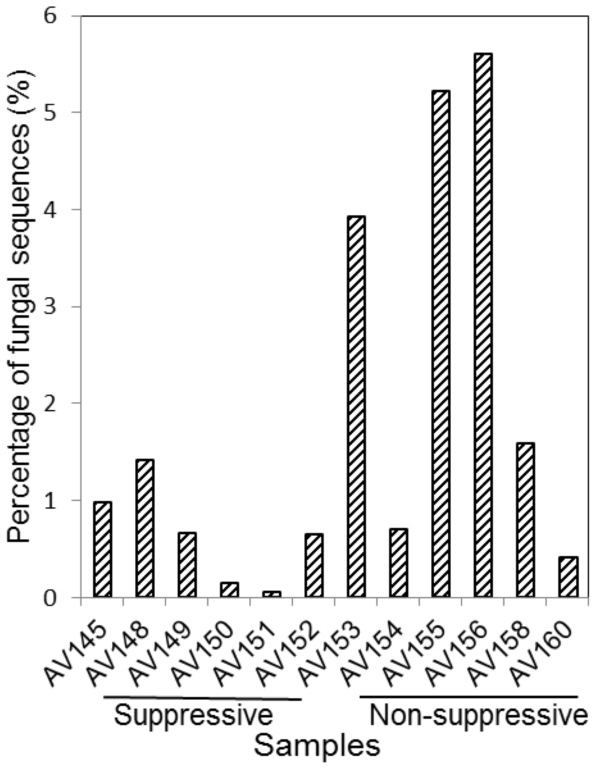

FIGURE S3. Relative abundance of *R. solani* AG8 sequences in fungal transcripts of metatranscriptomic libraries from the rhizosphere of wheat in suppressive (AV145-AV152) and non-suppressive (AV153-AV160) soil.
