# Supplementary material for: Comparative Metatranscriptomics of Wheat Rhizosphere Microbiomes in Disease Suppressive and Non-suppressive Soils for Rhizoctonia solani AG8
Source: Front Microbiol. 2018 May 4;9:859. doi: 10.3389/fmicb.2018.00859 (PMC5945926; doi:10.3389/fmicb.2018.00859)
Supplement: Supplementary file 11 [file Image_4.pdf]

A

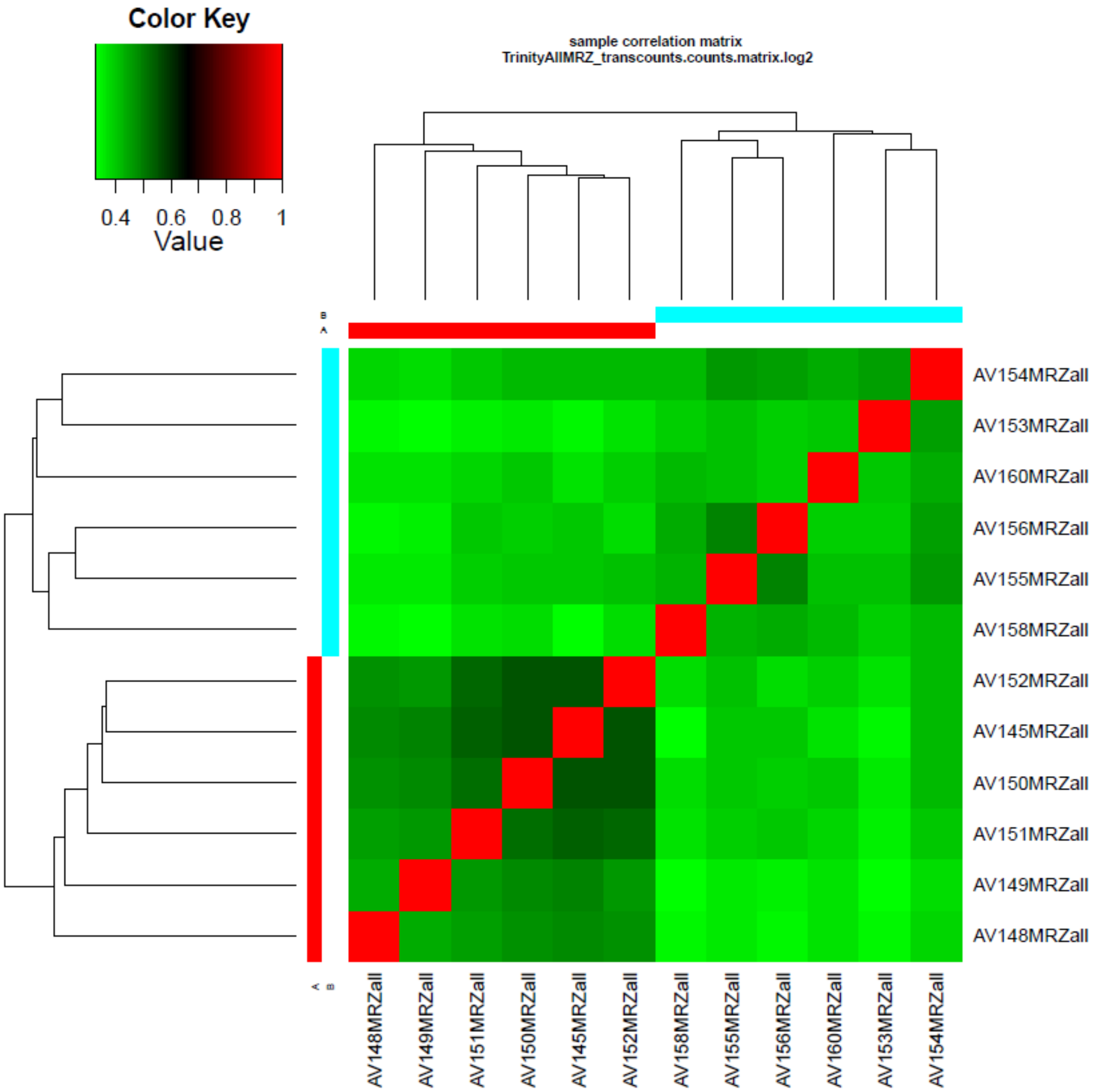

Color Key
0.4
0.6
0.8
1
Value
sample correlation matrix
TrinityAllMRZ_transcounts.counts.matrix.log2
B
A
AV154MRZall
AV153MRZall
AV160MRZall
AV156MRZall
AV155MRZall
AV158MRZall
AV152MRZall
AV145MRZall
AV150MRZall
AV151MRZall
AV149MRZall
AV148MRZall
AV148MRZall
AV149MRZall
AV151MRZall
AV150MRZall
AV145MRZall
AV152MRZall
AV158MRZall
AV155MRZall
AV156MRZall
AV160MRZall
AV153MRZall
AV154MRZall

B

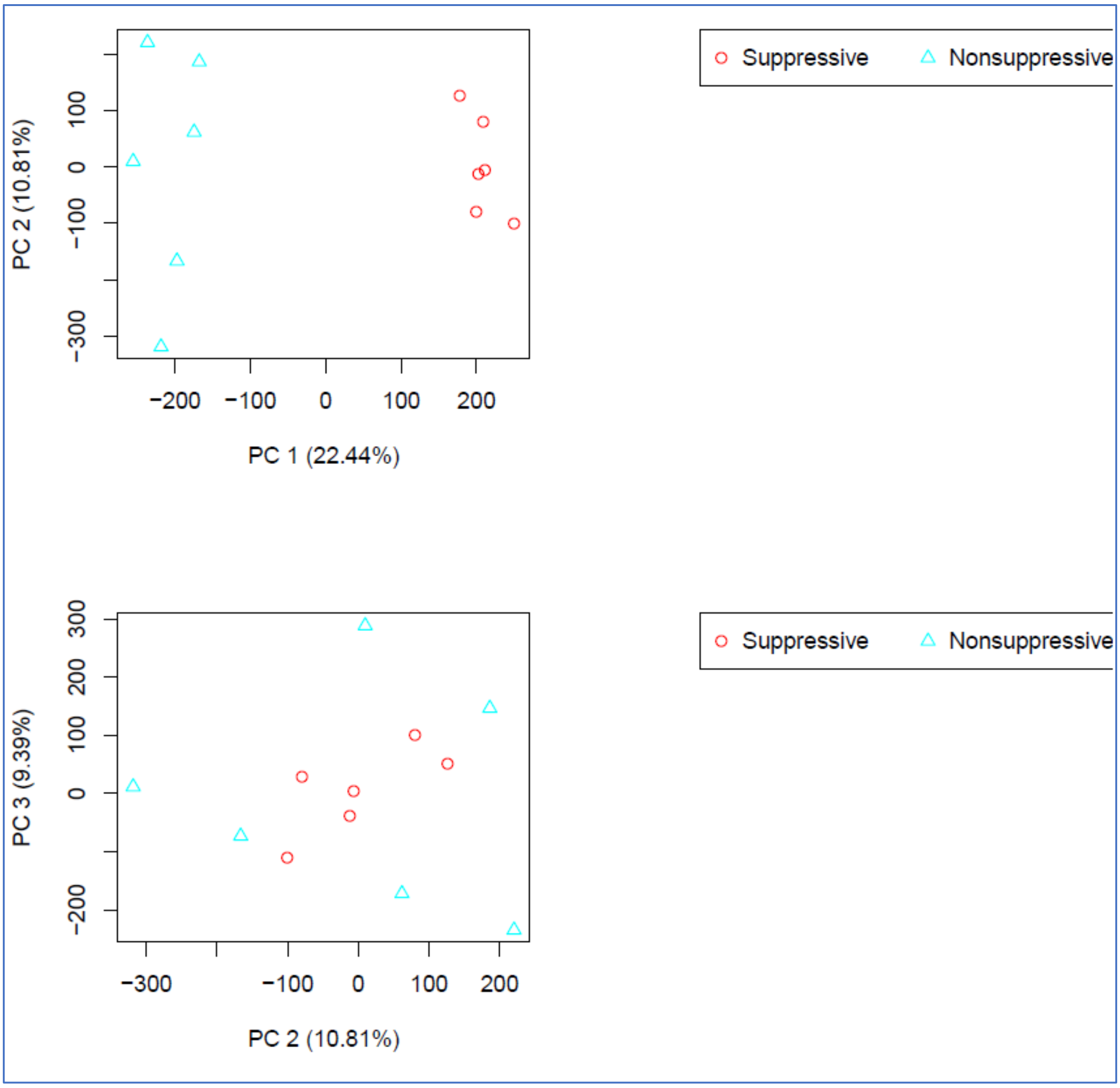

FIGURE S4. The similarity of biological replicate metatranscriptomes from the suppressive (AV145MRZall-AV152MRZall) and non-suppressive (AV153MRZall-AV160MRZall) field was examined by **A)** a Pearson correlation heatmap based on transcript expression values. Replicates are highly similarly when they are closer to a value of 1; and **B)** a principal component analysis (PCA) plot.
